# Genome Sequence of *Elaeagnus mollis*, the First Chromosome-Level Genome of the Family Elaeagnaceae

**DOI:** 10.1093/gbe/evab266

**Published:** 2021-12-02

**Authors:** Baoqing Ren, Dafu Ru, Luqin Chen, Na Duan, Yong Li, Jianwei Shi, Jianting Cao, Bingbing Liu

**Affiliations:** 1 Taiyuan Botanical Garden, China; 2 State Key Laboratory of Grassland Agro-Ecosystem, Institute of Innovation Ecology & School of Life Sciences, Lanzhou University, China; 3 Institute of Loess Plateau, Shanxi University, Taiyuan, China

**Keywords:** *Elaeagnus mollis*, oil tree, endangered

## Abstract

*Elaeagnus mollis* Diels (Elaeagnaceae) is a species of shrubs and/or dwarf trees that produces highly nutritious nuts with abundant oil and pharmaceutical properties. It is endemic to China but endangered. Therefore, to facilitate the protection of its genetic resources and the development of its commercially attractive traits we generated a high-quality genome of *E. mollis*. The contig version of the genome (630.96 Mb long) was assembled into 14 chromosomes using Hi-C data, with contig and scaffold N50 values of 18.40 and 38.86 Mb, respectively. Further analyses identified 397.49 Mb (63.0%) of repetitive sequences and 27,130 protein-coding genes, of which 26,725 (98.5%) were functionally annotated. Benchmarking Universal Single-Copy Ortholog assessment indicated that 98.0% of highly conserved plant genes are completely present in the genome. This is the first reference genome for any species of Elaeagnaceae and should greatly facilitate future efforts to conserve, utilize, and elucidate the evolution of this endangered endemic species.

Significance

We have established the first reference genome for any species of Elaeagnaceae. We believe it will facilitate studies on the processes of plant adaptation to harsh environments and disease resistance, as well as the breeding, conservation, and evolution of the species.

## Introduction

*Elaeagnus mollis* Diels (Elaeagnaceae) is a species of shrubs and/or dwarf trees with extremely distinctive winged fruits endemic to China (Qin and Gilbert 2007). Its seeds are highly nutritious and have high contents of oil that are both edible and have pharmaceutical properties (Liang et al. 2015). As a Tertiary relict plant, there are currently only four natural populations of *E. mollis*, narrowly distributed in southern parts of the Luliang Mountains and western parts of the Zhongtiao Mountains in Shanxi, and northern foot of the Qinling Mountain Range in Shaanxi (110°37′–111°56′E, 34°05′–36°05′N; Shangguan and Zhang 2001). Due to its extremely narrow range and limited population size, it is in the vulnerable category in the IUCN red list of threatened species, (http://www.iucnredlist.org/) and endangered species category of national key protected wild plants in China (http://rep.iplant.cn/). Unfortunately, recent habitat fragmentation caused by climate change and excessive commercial exploitation in recent years has caused severe contraction of its natural populations (Qin et al. 2010). Moreover, the *E. mollis* genome has not been previously sequenced, which has impeded both research and conservation efforts. Therefore, we have assembled a high-quality draft version of the *E. mollis* genome. For this, we extracted genomic DNA from leaf tissue, constructed three DNA libraries (paired-end, ONT [Oxford Nanopore Technology], and Hi-C), sequenced them, and generated about 236.7 Gb clean data (supplementary tables S1 and S2, Supplementary Material online). The final assembled contig version of the genome was about 630.95 Mb long, consisting of 131 contigs (N50 = 18.40 Mb). The draft genome was further refined using Hi-C data, and 620.77 Mb (98.4%) of the assembled bases were anchored onto 14 chromosomes, increasing the scaffold N50 to 38.86 Mb. To improve the quality of gene prediction and annotation, three independent approaches (ab initio prediction, homology searching, and reference-guided transcriptome assembly) were used for gene prediction, and five public protein databases (GenBank Non-Redundant [NR, 20200921], TrEMBL [202005], SwissProt [202005], eukaryotic orthologous groups [KOG, 20110125], gene ontology [GO, 20200615] and Kyoto Encyclopedia of Genes and Genomes [KEGG, 20191220]) were used for gene annotation. Finally, we identified 27,130 genes in the genome, 98.5% of which were successfully annotated for conserved functional motifs, and Benchmarking Universal Single-Copy Ortholog (BUSCO) evaluation indicated that it includes approximately 98.0% of highly conserved plant genes.

The first high-quality chromosome-level *E. mollis* genome assembled in this study provides a valuable genetic resource for further genomic, evolutionary, and conservation biology research, and should facilitate the protection of both *E. mollis* and other vulnerable species.

## Results and Discussion

### Genome Sequence and Assembly

To construct the *E. mollis* genome, we first generated 70.70 Gb MGI (MGISEQ2000 platform) paired-end reads (150 bp; supplementary table S1, Supplementary Material online). The estimated genome size was 551.52 Mb, with 1.10% heterozygosity, based on 17-mer analysis (supplementary fig. S1 and table S2, Supplementary Material online). We then used a combination of Nanopore long reads and Hi-C reads to produce the final sequenced and assembled *E. mollis* genome. In total, 2,650,109 ONT long reads (N50 length 28,412 bp, average length 20,564 bp) were generated from 54.50 Gb total sequence data (supplementary table S3, Supplementary Material online). De novo assembly yielded 131 contigs, with an N50 length of 18.40 Mb. The total length of the assembled genome was 630.95 Mb, somewhat larger than the genome size estimated by k-mer analysis (table 1).

**Table 1:** Statistics of the *Elaeagnus mollis* Genome and Gene Model Predictions

Parameter		Value
Contig assembly		
Total number of contigs		131
Assembly size (bp)		630,949,870
N50 (bp)		18,396,748
N90 (bp)		5,302,868
Largest contig (bp)		45,531,911
Scaffold assembly		
Total number of scaffolds		50
Assembly size (bp)		630,958,270
N50 (bp)		38,861,146
N90 (bp)		31,177,146
Largest scaffold (bp)		115,470,569
Annotation		
GC content		31.88%
Repeat density		63%
Number of protein-coding genes		27130
Average length of protein-coding genes (bp)		4381.18
Complete BUSCOs		1581 (97.96%)
Fragmented BUSCOs		10 (0.62%)
Missing BUSCOs		23 (1.43%)

**Fig. 1.**
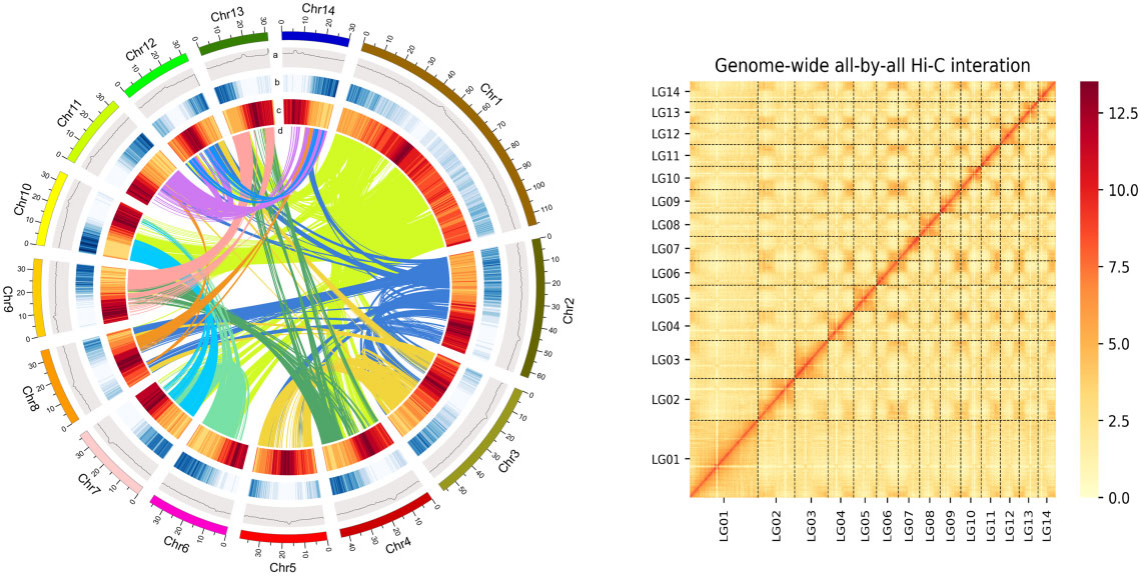
—The genome features of *Elaeagnus mollis*. (*A*) Circos plot showing features of the *E. mollis* genome. The concentric circles from the inner to outer show the GC density, gene density, repetitive sequence density, and collinearity; (*B*) Hi-C interaction matrices of the ordered scaffolds along the 14 pseudochromosomes.

The *E. mollis* assembly was further refined with valid interaction paired reads of Hi-C data, which were identified and retained (100.99 Gb) by HiC-Pro v2.8.1 (supplementary table S1, Supplementary Material online). Finally, 620.77 Mb (98.39%) of the contig sequences were anchored onto 14 chromosomes (fig. 1, supplementary table S4, Supplementary Material online). The final scaffold N50 was increased to 38.86 Mb and the longest scaffold was 115.47 Mb (table 1).

### Prediction and Functional Annotation of Protein-Coding Genes

Gene models in the *E. mollis* assembly were predicted using a combination of homology-based, reference-guided transcriptome assembly and ab initio gene approaches. Then, EVM software was employed to integrate the gene prediction results to produce a consensus gene set. To enhance the gene prediction quality, we removed miscoded genes and genes with transposable elements. Finally, we obtained a final gene set with 27,130 genes, and similar distributions of gene, coding, exon and intron lengths, and exon numbers, to those of other plants (supplementary fig. S2, Supplementary Material online).

#### Annotation of Repeat and Noncoding RNAs

We identified 397.49 Mb of nonredundant repetitive sequences in the *E. mollis* genome, representing 63% of the genome assembly. Long terminal repeat (LTR) retrotransponsons were the most abundant type, accounting for 41.46% of the whole genome (supplementary fig. S3 and table S5, Supplementary Material online), and Gypsy was the most abundant subfamily of the LTR family, accounting for 12.77% of the genome (supplementary table S5, Supplementary Material online), followed by Copia (11.65%, supplementary table S7, Supplementary Material online). We also identified 148 miRNAs, 796 transfer RNAs (tRNAs), 829 rRNAs, and 3,914 snRNAs with calculated average lengths of 123.37, 74.77, 668.99, and 106.81 bp, respectively.

### Genome Quality Assessment

To evaluate the genome assembly’s accuracy, short reads were mapped back to the consensus genome using BWA v0.7.12-r1039 (Burrows-Wheeler Aligner; Li and Durbin 2010) and an overall 99.75% mapping rate was obtained, covering 96.42% of the assembly. In addition, ONT reads were mapped back with Minimap2 (Li 2018), yielding an overall 99.72% mapping rate and 99.99% coverage of the assembly. These results suggest that our assembly contained nearly comprehensive genomic information. Furthermore, single-nucleotide polymorphisms (SNPs) were called and filtered using SAMtools v1.4 (Li et al. 2009), which identified 2,793,796 heterozygous SNPs, 7,566 homozygous SNPs, and 7,355 homozygous INDELs with 5× sequencing depth. The low rates of homozygous SNPs and INDELs (accounting for 0.001198% and 0.001164% of the assembled genome, respectively) corroborate the assembly’s high accuracy (99.997638%). Finally, the assembled *E. mollis* genome was divided into 10-kb nonoverlapping windows, and a scatter plot of the sequencing depth versus the GC-content based on 10-kb windows indicated that it had no contamination of foreign DNA (supplementary fig. S4, Supplementary Material online).

The completeness of gene regions was further assessed using the conserved core eukaryotic gene mapping approach (CEGMA; Parra et al. 2007), which indicated that our assembly captured 243 (97.98%) of conserved core eukaryotic genes, with 224 (90.32%) complete matches (supplementary table S6, Supplementary Material online).

Furthermore, we subjected the data to BUSCO analysis (Simão et al. 2015) using the embryophyta odb10 database (https://busco.ezlab.org/). Of 1,614 conserved plant genes, 97.96% had complete coverage in the genome (including 14.68% duplicates), 0.62% were fragmented, and only 1.43% were missing (supplementary table S7, Supplementary Material online). These data strongly indicate that our *E. mollis* genome assembly has high quality and validity for further investigation. BUSCO analysis was also applied to assess the completeness of these predicted genes, resulting in a BUSCO value of 95.54% (complete = 95.54%, single = 80.42%, duplicated = 15.12%, fragmented = 0.50%, missed = 3.97%, genes = 1,614; supplementary table S8, Supplementary Material online). In addition, indications of functions of 85.20% (23,114), 38.74% (10,510), 57.27% (15,536), 63.61% (17,257), and 98.32% (26,675) of the genes were obtained from searches against the Swiss-Prot, KEGG, KOG, GO, and NR databases, respectively (supplementary fig. S5 and table S9, Supplementary Material online). In total, 98.51% (26,725) of protein-coding genes were successfully annotated for conserved functional motifs or functional terms (supplementary fig. S5 and table S9, Supplementary Material online). These results clearly indicate that the annotated gene set of *E. mollis* genome is quite complete.

## Materials and Methods

### Plant Materials and DNA Extraction

Fresh young leaves were collected from an *E. mollis* plant growing naturally in Shanxi Province, China (112°28′30″E, 37°45′6″N) for high-quality genomic DNA sequencing. Total genomic DNA was isolated from the leaves with a QIAGEN Genomic kit (Cat. No. 13343, QIAGEN) according to the manufacturer’s standard protocols. Degradation and contamination of the extracted DNA were evaluated electrophoretically using 1% agarose gels. DNA purity was then assessed using an NanoDrop One UV-Vis spectrophotometer (Thermo Fisher Scientific, USA), and we obtained OD 260/280 ratios ranging from 1.8 to 2.0 and OD 260/230 ratios between 2.0 and 2.2. The DNA concentration of samples was also measured using a Qubit 3.0 Fluorometer (Invitrogen, USA).

### Library Construction and Sequencing

The genomic DNA was randomly fragmented using an M220 focused-ultrasonicator (Covaris, Woburn, MA, USA). For paired-end library preparation, the fragmented DNA (with an average size of 200–400 bp) were subjected to end-repair, 3′ adenylation, adapter-ligation, and PCR amplification, and the products were recovered using an AxyPrep Mag PCR clean-up kit. The double-stranded PCR products were heat-denatured and circularized by the splint oligo sequence. The single-stranded circle DNA fragments were formatted as the final library and qualified by QC. The qualified libraries were sequenced on an MGISEQ2000 platform. To check the reads’ reliability, MGI paired-end sequenced raw reads for the genomic survey were first filtered using the fastp v.0.20.0 preprocessor (Chen et al. 2018), with default settings, to remove low-quality reads, adapters, and reads containing poly-N.

The following types of low-quality reads were filtered out, those with ≥10% unidentified nucleotides (N); >10 nucleotides aligned to the adapter (allowing ≤10% mismatch); >50% bases with <5 Phred quality; and putative duplicates generated by PCR amplification in the library construction process (reads 1 and 2 of paired-end reads that were completely identical).

The fragments were then sequenced by Nextomics with a PromethION sequencer (Oxford Nanopore Technologies, UK). Output FAST5 files containing signal data were converted, via basecalling, to FASTQ format with Guppy v.3.2.2 + 9fe0a78 (Wick et al. 2019). The raw reads in fastq format with mean_qscore_template <7 were then filtered, resulting in pass reads (Cali et al. 2019). For Hi-C library construction, fresh *E. mollis* leaves were cut into 2 cm pieces and vacuum-infiltrated in nuclei isolation buffer (CTAB) supplemented with 2% formaldehyde. Crosslinking was stopped by adding glycine and further vacuum infiltration. Fixed tissue was then ground to powder and resuspended in nuclei isolation buffer to obtain a suspension of nuclei. The purified nuclei were digested with 100 units of DpnII and marked by incubation with biotin-14-dCTP. Biotin-14-dCTP was removed from nonligated DNA ends by exploiting the exonuclease activity of T4 DNA polymerase. The ligated DNA was sheared into 300–600 bp fragments, blunt-end repaired, A-tailed, then purified through biotin-streptavidin-mediated pull-down. Finally, the Hi-C libraries were quantified and sequenced using an Illumina Novaseq platform. In total, 673,270,118 paired-end reads of 150 bp were obtained from the Novaseq platform for the Hi-C library. Then, the raw Hi-C data were subjected to quality control using Hi-C-Pro as in previous studies. First, low-quality sequences (with quality scores <20), adaptor sequences, and sequences shorter than 30 bp were filtered out using fastp v.0.20.0 (Chen et al. 2018) then the clean paired-end reads were mapped to the draft assembled sequence using Bowtie2 v2.3.2 (Langmead and Salzberg 2012; -end-to-end –very-sensitive -L 30) to get the unique mapped paired-end reads. Valid interaction paired reads were identified and retained by HiC-Pro v2.8.1 (Servant et al. 2015) from unique mapped paired-end reads for further analysis. Invalid read pairs, including dangling-end, self-cycle, religation, and dumped products were filtered by HiC-Pro v2.8.1 (Servant et al. 2015).

Furthermore, leaves were collected from the same individual of *E. mollis*, and RNA-Seq reads were generated for genome annotation using the MGISEQ2000 platform. In total, 80.03 Mb of 150-bp paired-end reads were obtained after adapter trimming and quality filtering (supplementary table S1, Supplementary Material online).

### Estimation of the *E. mollis* Genome Size

Quality-filtered reads from the MGISEQ2000 platform were subjected to 17-mer frequency distribution analysis with Jellyfish v2.3.0 (Marçais and Kingsford 2011) to estimate the size and heterozygosity of the *E. mollis* genome. Based on the total number of k-mers (18,200,169,393), the *E. mollis* genome size was calculated using the following formula: genome size = k-mer_Number/Peak_Depth. Furthermore, the genome’s heterozygosity and repeat content were then estimated using simulations of Arabidopsis with different heterozygosity levels and the 17-k-mer frequency distribution.

### Genome Assembly

The 54.50 Gb ONT single-molecular long reads were assembled using NextDenovo v2.3.1 with a seed cutoff of 29 and 1 kb read length cutoff. Due to the high error rate of ONT raw reads, the original subreads were first self-corrected using the NextCorrect module to obtain consistent sequences (CNS reads). The CNS were then compared with the NextGraph module to capture correlations of CNS, which were used for preliminary genome assembly. To improve the assembly’s accuracy, the contigs were refined using Nextpolish v1.3.0 (Hu et al. 2020) with default parameters. To discard possibly redundant contigs and generate a final assembly, similarity searches were performed with the parameters “identity 0.8 -overlap 0.8.”

The *E. mollis* assembly was further refined with 100.99 Gb Hi-C data. Briefly, contigs/scaffolds of the *E. mollis* assembly were further clustered, ordered, and oriented onto chromosomes by LACHESIS (https://github.com/shendurelab/LACHESIS; Burton et al. 2013), with the following parameters: CLUSTER_MIN_RE_SITES = 100, CLUSTER_MAX_LINK_DENSITY = 2.5, CLUSTER NONINFORMATIVE RATIO = 1.4, ORDER MIN N RES IN TRUNK = 60, ORDER MIN N RES IN SHREDS = 60. Then placement and orientation errors exhibiting obvious discrete chromatin interaction patterns were corrected by manual adjusted.

### Identification of Repetitive Elements in *E. mollis*

Tandem Repeats Finder (TRF, v4.07b; Gary 1999) and GMATA v2.2 (Wang and Wang 2016) were employed to identify tandem repeats in the *E. mollis* genome. GMATA identifies simple repeat sequences and TRF recognizes all tandem repeat elements in genomes. For de novo prediction, MITE-hunter (Han and Wessler 2010), RepeatModeler v1.0.11 (http://www.repeatmasker.org/RepeatModeler.html; Price et al. 2005), and LTR_Finder v1.06 (Xu and Wang 2007) were utilized to construct a de novo repeat library. The obtained library was then aligned to TEclass Repbase (http://www.girinst.org/repbase; Abrusan et al. 2009) to classify the type of each repeat family. For further identification of the repeats throughout the genome, RepeatMasker v4.0.7 (Tarailo-Graovac and Chen 2009) was applied to search for known and novel TEs by mapping sequences against the de novo repeat library and Repbase (Jurka et al. 2005) TE library. Overlapping transposable elements belonging to the same repeat class were collated and combined.

### Gene Annotation

Three independent approaches (ab initio prediction, homology searching, and reference-guided transcriptome assembly) were used to predict genes in the repeat-masked genome. In detail, GeMoMa v1.6.1 (Jens et al. 2016) was used to align the homologous peptides from related species (*Arabidopsis thaliana*, *Cannabis sativa*, *Prunus mume*, and *Ziziphus jujuba*) with the assembly then gene structure information was obtained for homolog prediction. For RNA-seq-based gene prediction, filtered mRNA-seq reads were aligned to the reference genome using STAR v2.7.3a (Dobin et al. 2013). The transcripts were then assembled using StringTie v1.3.4d (Pertea et al. 2015) and open reading frames (ORFs) were predicted using PASA v2.3.3 (Haas et al. 2008). We also generated a training set for the de novo prediction. Augustus v3.3.1 (Mario et al. 2006) and GlimmerHMM (Majoros et al. 2004) with default parameters were then utilized for ab initio gene prediction with the training set. Finally, EvidenceModeler (EVM, v1.1.1; Haas et al. 2008) was used to produce an integrated gene set, from which genes with TEs were removed using the TransposonPSI package (http://transposonpsi.sourceforge.net/; Urasaki et al. 2016) and miscoded genes were further filtered. Untranslated regions (UTRs) and alternative splicing regions were determined using PASA based on RNA-seq assemblies. We retained the longest transcripts for each locus, and regions outside of the ORFs were designated UTRs.

Gene function information, motifs, and domains of predicted protein-coding genes were acquired by searching against five protein/function databases. InterProScan v5.36 (Zdobnov and Rolf 2001) was used for comprehensive annotation of predicted protein-coding genes, including annotation of GO terms, protein motifs and domains, functional classifications, protein family identification, transmembrane topologies, and predicted signal peptides. KaaS (https://www.genome.jp/kegg/kaas/) was used to search the KEGG database (Ogata et al. 1999) for KO terms. BLASTP v.2.7.1 (Altschul et al. 1997) was used for searches against the Swiss-Prot (Bairoch and Apweiler 2000), NR, and KOG (Galperin et al. 2015) databases with an *E* value cutoff of 1e-5. The results were integrated from the best hits of these database searches.

### Annotation of Noncoding RNAs

To identify noncoding RNA sequences in the genome, two strategies were used: database searching and model-based prediction. tRNAs were predicted using tRNAscan-SE v2.0 (Lowe and Eddy 1997) with eukaryote parameters. MicroRNA, rRNA, small nuclear RNA, and small nucleolar RNA sequences were detected using INFERNAL v1.1.2 (Nawrocki and Eddy 2013) to search the Rfam (Griffiths-Jones 2004) database. The rRNAs and their subunits were predicted using RNAmmer v1.2 (Lagesen et al. 2007).

## Supplementary Material

Supplementary data are available from *Genome Biology and Evolution* online.

## Supplementary Material

evab266_Supplementary_Data
