# Supplementary material for: Genome Sequence of Elaeagnus mollis, the First Chromosome-Level Genome of the Family Elaeagnaceae
Source: Genome Biol Evol. 2021 Dec 2;13(12):evab266. doi: 10.1093/gbe/evab266 (PMC8691057; doi:10.1093/gbe/evab266)

**Supplementary information**

**Table S1. Characteristics of NGS data.**

| Library type | Platform | Total reads | Total bases | Clean reads | Clean bases |
| --- | --- | --- | --- | --- | --- |
| Short reads | MGISEQ2000 | 518,402,930 | 77,760,439,500 | 506,647,748 | 70,700,484,894 |
| RNA-seq | MGISEQ2000 | 80,197,702 | 12,029,655,300 | 80,028,594 | 11,146,369,656 |
| Hi-C | Illumina NovaSeq | 673,270,118 | 100,990,517,700 | 669,841,612 | 100,375,699,160 |

**Table S2. Characteristics of ONT long reads.**

| Read_type | Read bases / bp | Read Number | Read length (max) / bp | Read length (mean) / bp | Read length (N50) / bp |
| --- | --- | --- | --- | --- | --- |
| Subreads | 54,497,503,697 | 2,650,109 | 170,138 | 20,564 | 28,412 |

**Table S3 Statistics of 17-mers obtained from the E. mollis genome.**

| k-mer | No. of k-mers | k-mer depth | Genome size (bp) | Heterozygous ratio (%) |
| --- | --- | --- | --- | --- |
| 17 | 18,200,169,393 | 33 | 551,520,284 | 1.10 |

**Table S4 Characteristics of the 14 *E. mollis* chromosomes.**

| **Chr** | **Size (bp)** | **No. of scaffolds** |
| --- | --- | --- |
| LG01 | 115,469,569 | 11 |
| LG02 | 62,725,179 | 4 |
| LG03 | 56,614,137 | 4 |
| LG04 | 43,634,681 | 12 |
| LG05 | 38,860,346 | 9 |
| LG06 | 36,689,038 | 2 |
| LG07 | 36,476,563 | 2 |
| LG08 | 34,989,334 | 8 |
| LG09 | 34,836,673 | 5 |
| LG010 | 34,164,633 | 10 |
| LG011 | 32,845,666 | 4 |
| LG012 | 32,006,992 | 4 |
| LG013 | 31,175,446 | 18 |
| LG014 | 30,278,703 | 5 |
| Total | 620,766,960 | 98 |

**Table S5. Summary of repetitive elements annotated in the genome of** ***E. mollis*.**

| Element type | No. of elements | Length occupied (bp) | Percentage of genome (%) |
| --- | --- | --- | --- |
| LTR | 477,565 | 261,615,542 | 41.46 |
| LINE | 115,809 | 29,417,615 | 4.66 |
| SINE | 20,043 | 2,244,244 | 0.36 |
| DNA | 330,023 | 77,304,157 | 12.25 |
| MITE | 11,429 | 2,398,718 | 0.38 |
| RC | 4,447 | 655,563 | 0.10 |
| Tandem repeats | 106,091 | 4,919,766 | 0.78 |
| Simple repeats | 1,288 | 213,922 | 0.03 |
| Unknown | 96,292 | 18,515,453 | 2.93 |
| Other | 671 | 200,786 | 0.03 |
| Low complexity | 10 | 971 | 0.00 |
| Total | 1,163,668 | 397,486,737 | 63.00 |

**Table S6. Summary results of CEGMA assessment of *E. mollis* genome.**

| Type | complete | | Complete + partial | |
| --- | --- | --- | --- | --- |
|  | Prots | %completeness | Prots | %completeness |
| Total | 224 | 90.32 | 243 | 97.98 |
| Group1 | 59 | 89.39 | 66 | 100.00 |
| Group2 | 47 | 83.93 | 53 | 94.64 |
| Group3 | 55 | 90.16 | 60 | 98.36 |
| Group4 | 63 | 96.92 | 64 | 98.46 |

**Table S7. BUSCO notation assessment of the *E. mollis* genome.**

| Type | Number | Percent (%) |
| --- | --- | --- |
| Complete BUSCOs (C) | 1,581 | 97.96 |
| Complete and single-copy BUSCOs (S) | 1,344 | 83.27 |
| Complete and duplicated BUSCOs (D) | 237 | 14.68 |
| Fragmented BUSCOs (F) | 10 | 0.62 |
| Missing BUSCOs (M) | 23 | 1.43 |
| Total BUSCO groups searched | 1,614 | 100.00 |

**Table S8. BUSCO notation assessment of *E. mollis* predicted genes.**

| Type | Number | Percent (%) |
| --- | --- | --- |
| Complete BUSCOs (C) | 1,542 | 95.54 |
| Complete and single-copy BUSCOs (S) | 1,298 | 80.42 |
| Complete and duplicated BUSCOs (D) | 244 | 15.12 |
| Fragmented BUSCOs (F) | 8 | 0.50 |
| Missing BUSCOs (M) | 64 | 3.97 |
| Total BUSCO groups searched | 1,614 | 100.00 |

**Table S9. Summary of functional annotation of protein-coding genes in the *E. mollis* genome.**

| Type | | Number | Percent (%) |
| --- | --- | --- | --- |
| Annotation | Swiss-Prot | 23,114 | 85.20 |
|  | KEGG | 10,510 | 38.74 |
|  | KOG | 15,536 | 57.27 |
|  | GO | 17,257 | 63.61 |
|  | NR | 26,675 | 98.32 |
| Total | Annotated | 26,725 | 98.51 |
|  | Gene | 27130 | - |

**Fig. S1 17-mer distribution in** **the *E.mollis* genome.**


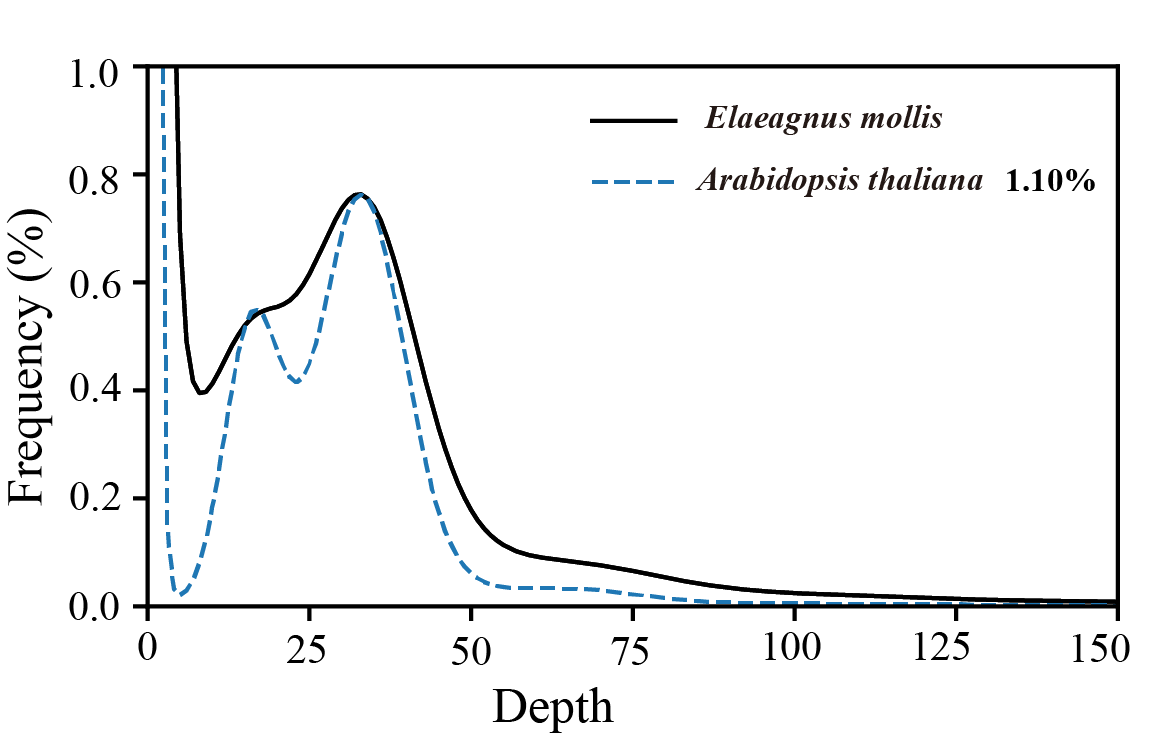


**Fig. S2 Comparison of structural characteristics of *E. mollis* genes with those in other genomes. (a) CDS length distribution, (b) exon length distribution, (c) exon number distribution, (d) gene length distribution, (e) intron length distribution, (f) intron number distribution. The x-axis indicates length or number and the y-axis the density of genes.**


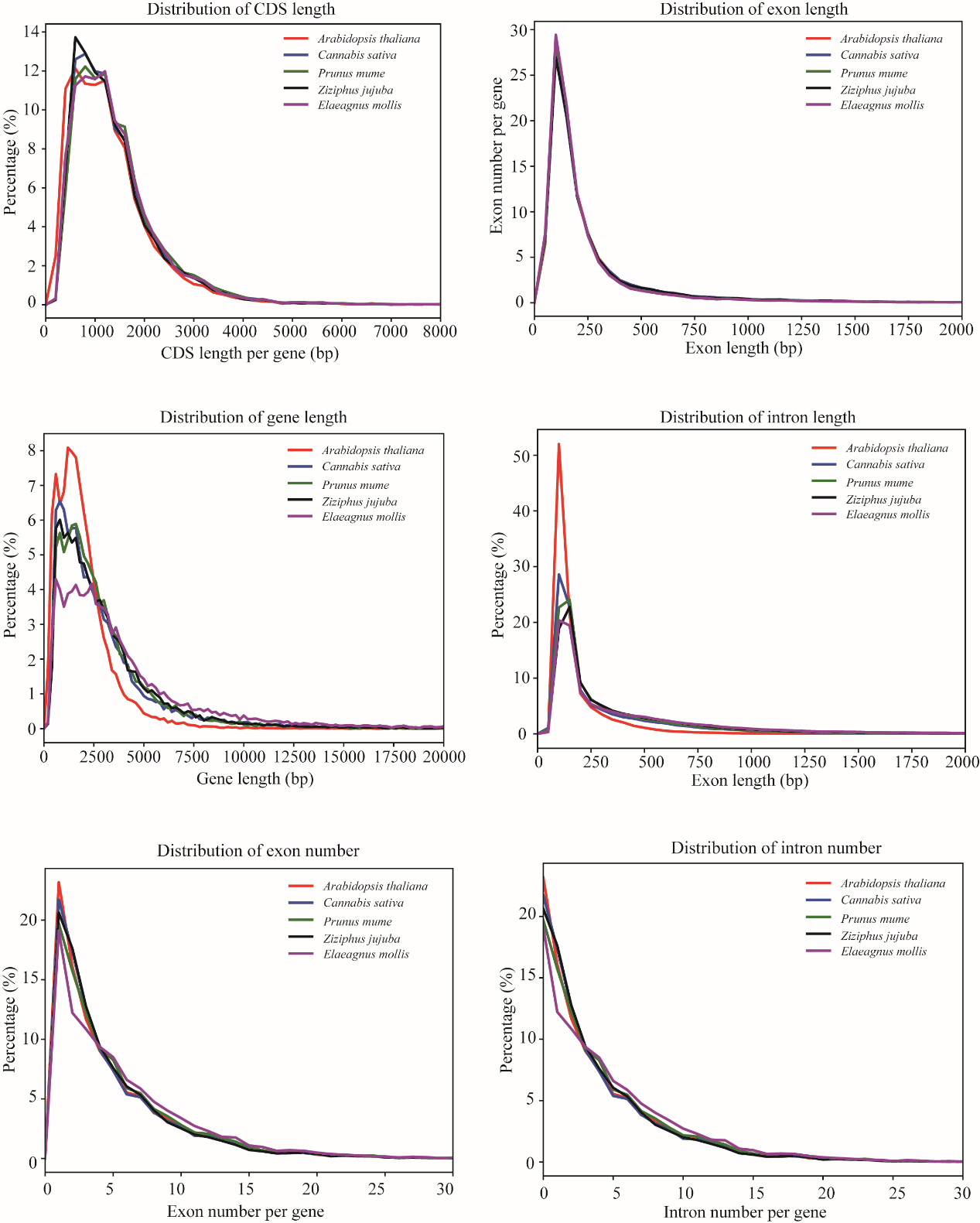


**Fig. S3 Sequences divergence rate of repeats annotated in *E.mollis*. The x-axis indicates the sequence divergence rate of repeats, and the y-axis the percentage of repeat sequences in the genome.**

**
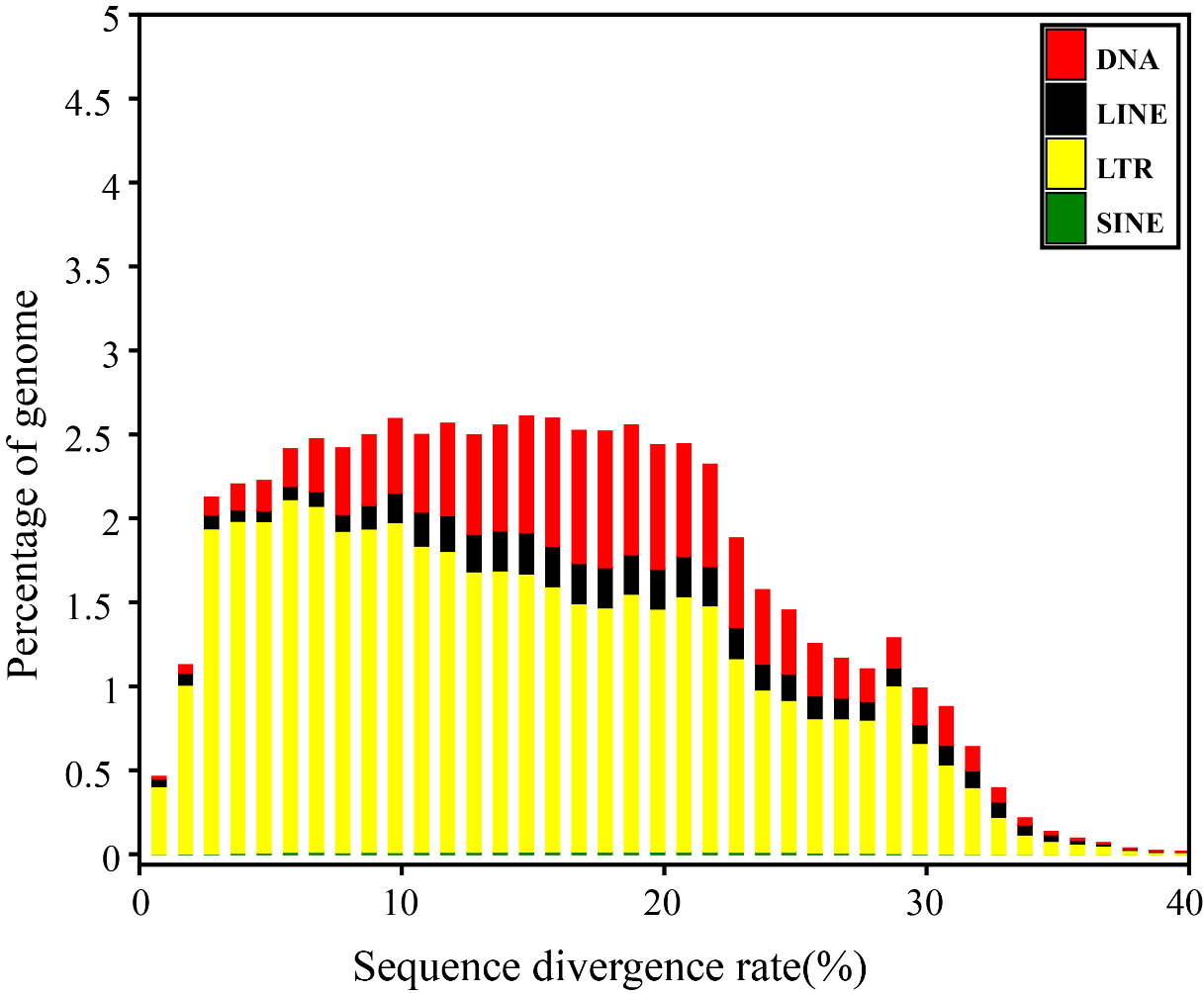
**

**Fig. S4 Distribution of GC depth, GC values and depth calculated with a 10 kb window size. The x-axis indicates GC content and y-axis the depth.**

**
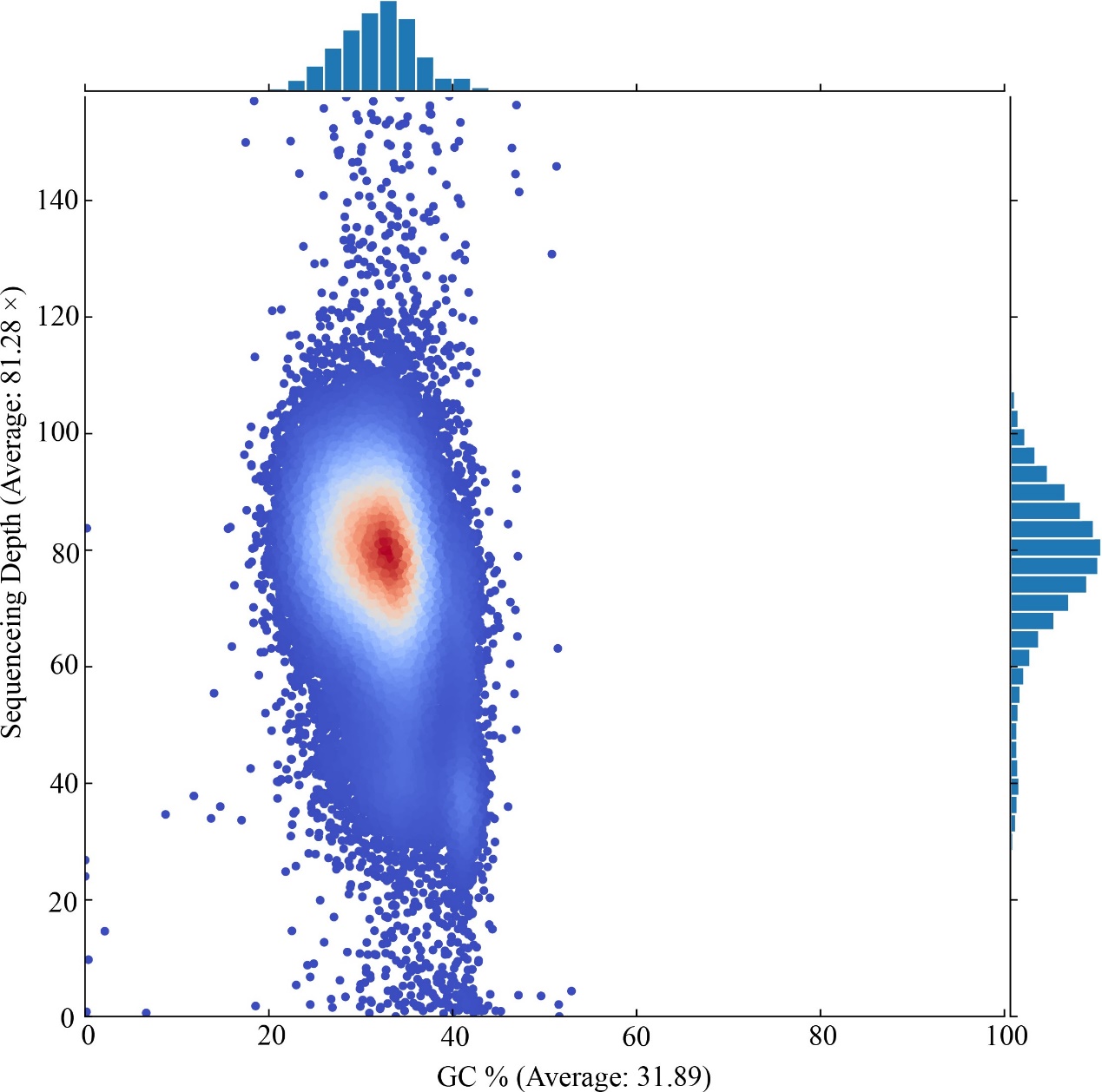
**

**Fig. S5 Venn diagram of numbers of genes in the *E. mollis* genome functionally annotated using indicated public databases.**


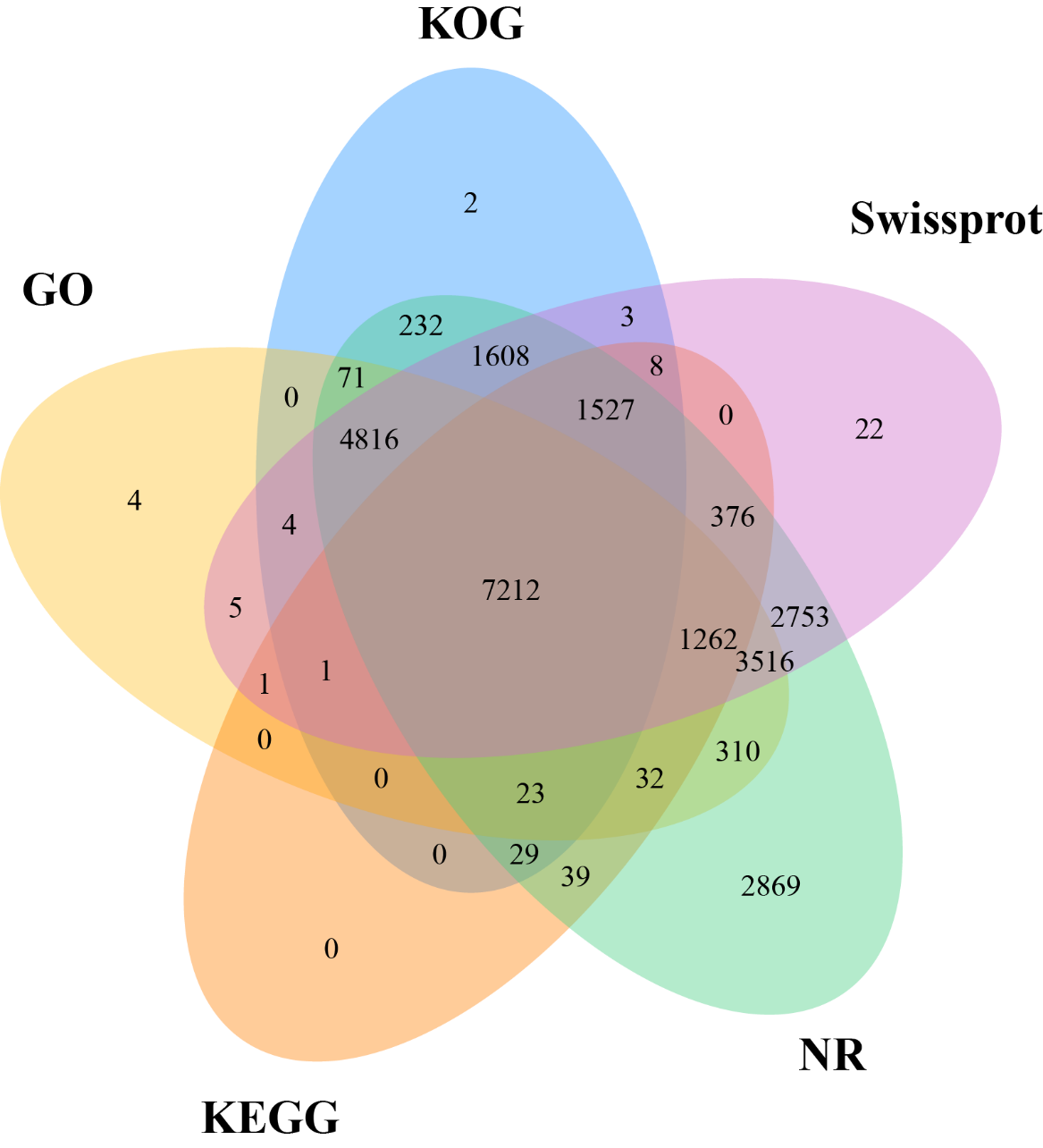

Supplement: evab266_Supplementary_Data [file evab266_supplementary_data.docx]
